# Thermal–hydraulic analysis of the coil test facility for CFETR

**DOI:** 10.1186/s40064-016-3706-z

**Published:** 2016-12-01

**Authors:** Yong Ren, Xiaogang Liu, Junjun Li, Zhaoliang Wang, Lilong Qiu, Shijun Du, Guoqiang Li, Xiang Gao

**Affiliations:** Institute of Plasma Physics, Hefei Institutes of Physical Science, Chinese Academy of Sciences, PO Box 1126, Hefei, 230031 Anhui People’s Republic of China

**Keywords:** Cable-in-conduit conductor (CICC), CFETR, Quench, Superconducting magnet, Thermal–hydraulic behavior

## Abstract

**Background:**

Performance test of the China Fusion Engineering Test Reactor (CFETR) central solenoid (CS) and toroidal field (TF) insert coils is of great importance to evaluate the CFETR magnet performance in relevant operation conditions. The superconducting magnet of the coil test facility for CFETR is being designed with the aim of providing a background magnetic field to test the CFETR CS insert and TF insert coils.

**Results:**

The superconducting magnet consists of the inner module with Nb_3_Sn coil and the outer module with NbTi coil. The superconducting magnet is designed to have a maximum magnetic field of 12.59 T and a stored energy of 436.6 MJ. An active quench protection circuit and the positive temperature coefficient dump resistor were adopted to transfer the stored magnetic energy.

**Conclusions:**

The temperature margin behavior of the test facility for CFETR satisfies the design criteria. The quench analysis of the test facility shows that the cable temperature and the helium pressure inside the jacket are within the design criteria.

## Background

PERFORMACNE test of the China Fusion Engineering Test Reactor (CFETR) central solenoid (CS) and toroidal field (TF) insert coils is of great importance to evaluate the CFETR magnet performance in relevant operation conditions (Ren et al. 2015a). A superconducting magnet of the coil test facility for CFETR magnet is being designed. The superconducting magnet consists of two parts, i.e. the inner part with Nb_3_Sn coil and the outer part with NbTi coil. Both coils are designed based on the Cable-In-Conduit Conductor (CICC) technology. The superconducting magnet has a stored energy of 436.6 MJ and a maximum magnetic field of 12.59 T. The superconducting magnet will be cooled with supercritical helium at 4.5 K inlet temperature. During the operation, the AC losses due to changing magnetic fields and the heat leak from the environment will increase the operating temperature and minimize the temperature margin (Ren et al. 2012). Therefore, the temperature margin behavior of the superconducting magnet needs to be analyzed in relevant operating conditions. Once the operating temperature exceeds the current sharing temperature, a large hot spot temperature in the normal zone region can be developed. It is very important to make every effort to avoid quench of the superconducting magnet from the electromagnetic and heat disturbance. However, the appropriate quench design is mandatory to avoid the severe failure due to the overheating of the cable and the overstressing of the jacket during a quench (Wang et al. 2004; Meuris et al. 2010; Takahashi et al. 2006). In addition, the appropriate quench protection circuit is required to distinguish between quench signal and noise (Gaio et al. 2013; Takahashi et al. 2005; Martovetsky and Radovinsky 2006; Lacroix et al. 2013). The parametric analysis on the hot spot temperature and helium pressure behavior is required to understand the influence of the initial some initial thermal–hydraulic parameters for the quench analysis (Nicollet et al. 2013).

To reduce the quench voltage of the coil terminals and to accelerate the current decay during a quench, the ST-08 stainless steel with positive temperature coefficient will be adopted as the material of dump resistor (Ren et al. 2015b). The 1-D GANDALF code was used to analyze the temperature margin and quench propagation behavior (Bottura 1996).

In this paper, the temperature margin behavior and the quench propagation behavior of the superconducting magnet are described.

## Results

### Description of the superconducting magnet

**Fig. 1:**
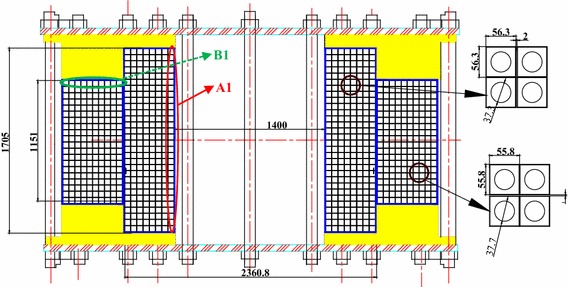
Cross section of the winding pack of the superconducting magnet

**Fig. 2:**
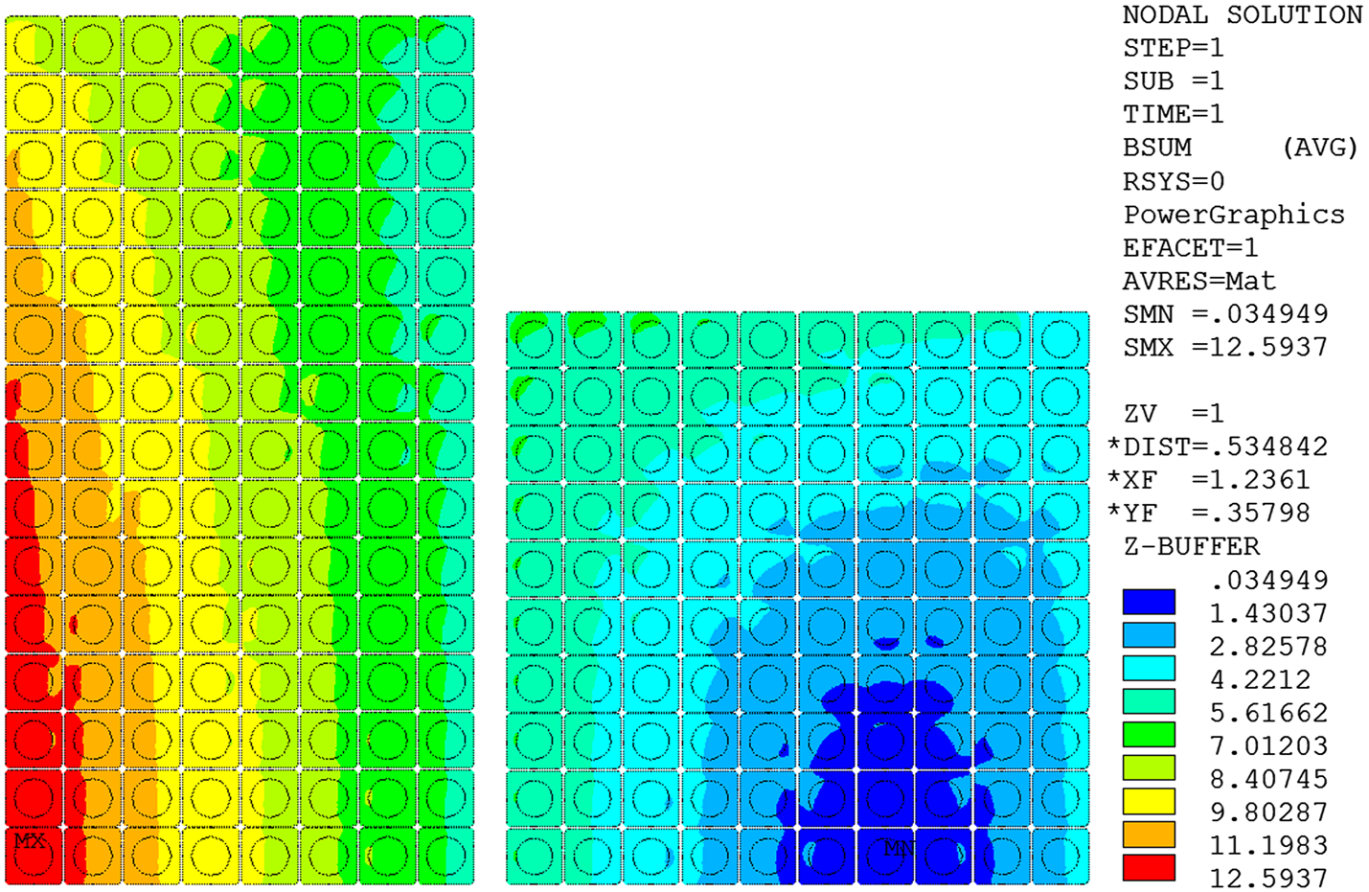
Magnetic field distribution of the superconducting magnet at 56 kA (Unit: T)

The superconducting magnet consists of the inner module with Nb_3_Sn coil and the outer module with NbTi coil. The Nb_3_Sn cable is made of 864 Nb_3_Sn and 432 copper strands inserted into a round-in-square modified 316LN stainless steel jacket with low carbon content to form a CICC conductor. The NbTi cable is made of 1440 NbTi strands inserted into a round-in-square 316L stainless steel jacket to form a CICC conductor. The Nb_3_Sn coil, which is layer-wound winding, has eight layers with eight cooling channels. The NbTi coils are pancake wound to minimize the length of the cooling channel. There are ten cooling channels for NbTi coils; each cooling channel has two pancakes. Table 1 lists the design parameters of the superconducting magnet. Figure 1 shows the cross section of a winding pack of the superconducting magnet. Figure 2 shows the magnetic field distribution of the superconducting magnet.

**Table 1:** Design parameters of the superconducting magnet

Superconductor	Nb_3_Sn	NbTi
Jacket	316LN	316L
Inner diameter (mm)	1.4000	2.3808
Outer diameter (mm)	2.3608	3.5288
Height (mm)	1.7050	1.1510
Turn insulation (mm)	1.0	1.0
Layer/pancake insulation (mm)	2.0	1.0
Layer/Pancake	8	20
Turns per layer or pancake	30	10
Void fraction in the CICC	0.3	0.34
Current (kA)	56	
Inductance (H)	0.2784	
Stored energy (MJ)	436.6	
Maximum field (T)	12.59	5.455

The Nb_3_Sn CICC conductors are cooled with the forced flow supercritical helium at 0.55 MPa pressure, 12 g/s mass flow rate and 4.5 K temperature at the coil inlet. The NbTi coils are cooled with supercritical helium at inlet pressure of 0.55 MPa, mass flow rate of 8 g/s and 4.5 K temperature at the inlet. The hydraulic parameters of the Nb_3_Sn and NbTi CICCs for the superconducting magnet can be shown in references (Ren et al. 2015a; ITER 2009).

**Fig. 3:**
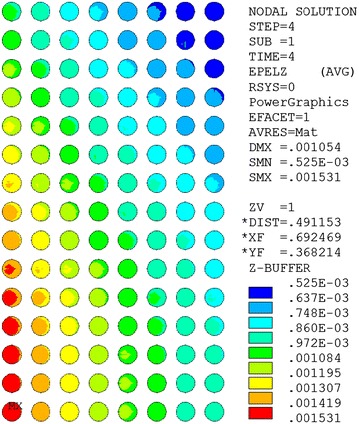
Hoop strain distribution of the superconducting cables at 56 kA

The scaling law and the scaling parameters of the Nb_3_Sn superconductor can be shown in Ref. (ITER 2009; Godeke et al. 2006). The effective filament diameter is 30 μm for the Nb_3_Sn strand (Bottura 2000). The longitudinal strain of the Nb_3_Sn strand is mainly composed of the thermal strain from the thermal contraction and the strain from the magnetic loading. The thermal strain of the Nb_3_Sn strand in a CICC with 316LN stainless steel jacket was assumed as −0.664% (ITER 2009). The cables in the CICCs are assumed to be fully bonded to the inner surface of the jacket for the mechanical behavior analysis. The 316LN stainless steel jacket with isotropic material properties and the insulation material with orthotropic material properties are used for the mechanical behavior analysis, which can be described in Ref. (Jong and al 2009). The effective material properties in the cable regions, which consists of Young’s modulus, shear modulus, thermal contraction and Poisson ratio, can be obtained with the finite element methods based on the homogeneous theory (Kaminski and Schrefler 2000). A linear elastic analysis using the relevant stress–strain model is performed to analyze the strain of the cable. The strain generated by the magnetic force is shown in Fig. 3. The critical current density of the NbTi superconductor can be obtained by using the single pinning model (Bottura 2000). The scaling law and the scaling parameters of the critical current density in NbTi superconductor can be shown in relevant expressions (Bottura 2000; Zani et al. 2005). The effective filament diameter is about 8 μm for the NbTi strand. It is hard to accurately evaluate the coupling time constant of the CICC conductors. The coupling time constants of the CICC conductors are usually dependent on the local magnetic forces, the load cycle process, void fraction, cable layout, aspect ratio, coating material of the cable, and the magnet ramp rate, etc. (Bruzzone et al. 2006; Bruzzone et al. 2009; Hamada et al. 2004; Ilyin et al. 2010; Cau and Bruzzone 2010; Cau et al. 2009). For simplicity, the coupling time constants with nτ values of the Nb_3_Sn and NbTi CICC conductors were selected as 0.075 and 0.15 s for evaluating AC losses respectively (ITER 2009). The pressure drop in the central channel and the bundle for the CICC using the relevant expressions can be described in (1–3) (Cau et al. 2009; Hamada et al. 2002; Nicollet and al 2014).

1 $$\frac{dp}{dx} = - 2\rho \frac{f}{{D_{h} }}v\left| v \right|$$

2 $$f_{bundle} = \frac{1}{{4v_{f}^{0.742} }}\left(\frac{19.5}{{\text{Re}^{0.7953} }} + 0.0231\right)$$

3 $$f_{central} = 0.36 \times \frac{1}{{\text{Re}^{0.04} }} \times 0.25$$

where *dp/dx* is the pressure gradient, *f* is the friction factor, *f*_bundle_ and *f*_central_ are friction factors of the bundle and the central channel, *ρ* is the density of the helium, *v* is the flow speed, *v*_*f*_ is the void fraction, Re is the Reynolds number, *D*_h_ is the hydraulic diameter.

## Thermal–hydraulic analysis of the superconducting magnet

### Current sharing temperature of the superconducting magnet

**Fig. 4:**
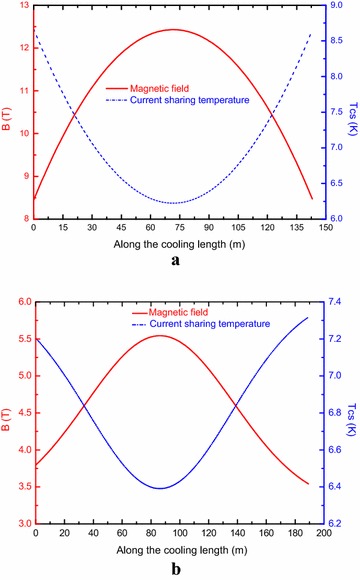
Magnetic field and current sharing temperature at 56 kA along the cooling length of **a** the A1 channel and **b** the B1 channel

The maximum magnetic field is located at the innermost layer for the Nb_3_Sn coil. The temperature margin and quench propagation behavior of the innermost layer of the Nb_3_Sn coil were analyzed. For the NbTi coils, the temperature and quench behavior of the top channel was analyzed due to the lowest value of the minimum temperature margin is located at the top and bottom channels symmetrically. Here, we refer the innermost layer of the Nb_3_Sn coil and the top channel of the NbTi coil as A1 and B1 channels, as shown in Fig. 1. Figure 4 shows the current sharing temperature of the A1 channel and the B1 channel when the operating current reaches the rated current. The minimum current sharing temperature are about 6.3 and 6.4 K for both channels.

**Fig. 5:**
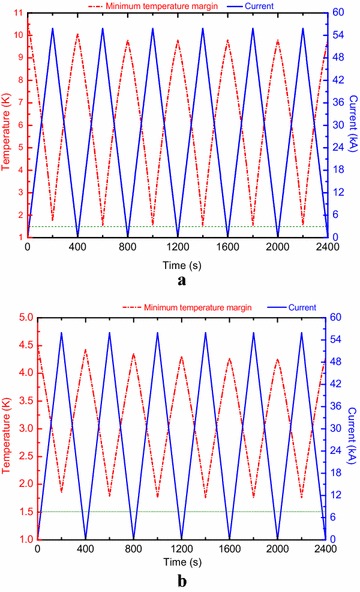
Minimum temperature margin and current evolution as functions of time for **a** the A1 channel and **b** the B1 channel

**Fig. 6:**
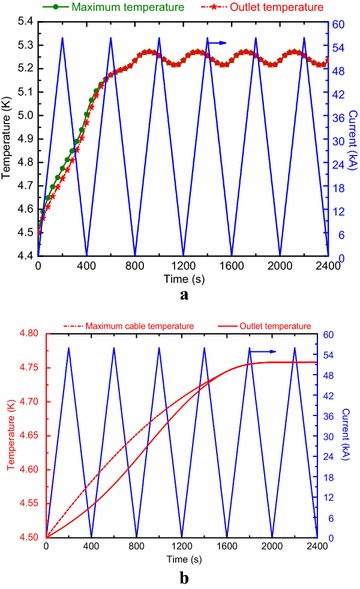
Maximum temperature, outlet temperature and current evolution as functions of time for **a** the A1 channel and **b** the B1 channel

The superconducting magnet has a chance to operate in a cyclic operation to evaluate the conductor performance. Here, we selected a typical cyclic operation mode when the magnet is linearly ramped up to the rated field and then ramped down to zero field, cycle after cycle. The ramp rate of 280 A/s was firstly assumed in this case. Figures 5 and 6 show the maximum cable temperature, outlet temperature and minimum temperature margin evolution as functions of time for both channels. The analysis results are shown that the lowest values of the minimum temperature margin for the A1 and B1 channels are 1.50 and 1.70 respectively.

**Fig. 7:**
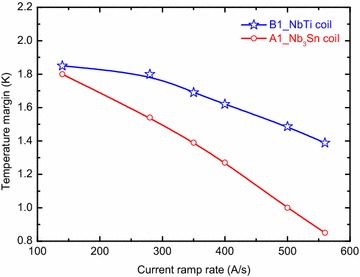
The lowest value of the minimum temperature margin vs current ramp rate for cyclic operation

A parametric analysis was performed to evaluate the minimum temperature margin sensitivity to the current ramp rate for both channels. Figure 7 shows the minimum temperature margin as a function of the ramp rate for cyclic operation. It is shown that the continuous cyclic operations can be allowed for the current ramp rate below 280 A/s. With increasing the current ramp rate, the minimum temperature margin will drop below 1.0 K for the current ramp rate of 500 A/s.

### Quench analysis of the superconducting magnet

The superconducting magnet will store a large magnetic energy of about 436.6 MJ at 56 kA. To avoid the overheating from the hot spot temperature, an appropriate quench protection is required. The quench protection circuit is composed of the DC power supply, quench detection circuit, coil isolation amplifiers, dump resistor, diode stack, breaker control system, switch control system and emergency dump breakers etc. The quench detection circuit will be used to monitor the voltage of the superconducting magnet. The high speed DC circuit breakers are used to protect the superconducting coils in case of a quench. The input power will be interrupted by opening the emergency dump circuit breakers.

The quench propagation behavior was analyzed with the 1 D Gandalf code, together with the adiabatic hot spot temperature criterion. The allowable maximum hot spot temperature is about 150 K with the 1-D Gandalf code. The allowable maximum hot spot temperature is about 250 K by considering only the heat capacity of the cable for the adiabatic hot spot temperature criterion. The initial quench triggering point can be taken place in Nb_3_Sn coil or NbTi coil. So, the quench propagation behavior of the A1 channel and B1 channel needs to be analyzed.

**Fig. 8:**
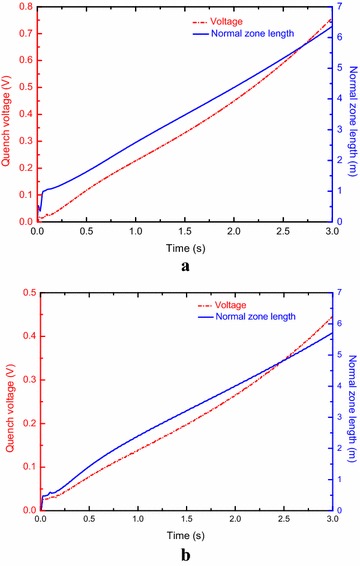
Normal zone length and quench voltage evolution as functions of time for **a** the A1 channel and **b** the B1 channel

The normal zone length and quench voltage can be obtained with the 1 D GANDALF code. To trigger a quench, a rectangular heat input (1 m, 0.1 s) was exerted into the cable to drive the cable into the normal state. The triggered energy adopted is about 2 times the energy needed to cause an irreversible quench. Figure 8 shows the normal zone length and quench voltage as functions of time for the A1 and B1 channels. It takes about 1.80 s to reach the quench voltage of 0.4 V for the A1 channel. It takes about 2.78 s to reach the quench voltage of 0.4 V for the B1 channel. To open the circuit breaker, it will take about 0.5 s. As a first step, the adiabatic hot spot temperature criteria, together with the quench voltage behavior calculated by the 1 D GANDALF code was adopted to obtain the maximum holding time. The holding time represents the period between quench detection and breaker opening. The total equivalent thermal time constant of the A1 channel and B1 channel are about 9.8 and 8.2 s respectively.

The discharge time constant is selected as 2.8 s for the superconducting magnet. The maximum holding time can be obtained as 6.1 s by taking into account the threshold voltage of 0.4 V if the quench originated from the A1 channel. The maximum holding time can be obtained as 3.52 s by taking into account the threshold voltage of 0.4 V and the discharge time constant of 2.8 s if the quench originated from the B1 channel. As shown in Ref. (Martovetsky et al. 2016), the cable temperature differs from the jacket temperature as a result of the quench propagation with delay. Therefore, a relatively less holding time was selected for limiting the hot spot temperature of the cable. The threshold voltage and holding time can be designed as 0.4 V and 2.0 s respectively.

**Fig. 9:**
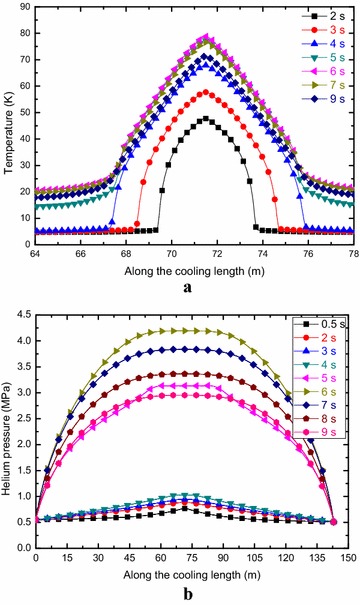
Cable temperature (**a**) and Helium pressure (**b**) evolution along the cooling length as functions of time for the A1 channel

Figure 9 shows the cable temperature and helium pressure evolution along the cooling length of the A1 channel as functions of time with the holding time of 2.0 s, initial disturbance length of 1 m and quench threshold voltage of 0.4 V. The maximum cable temperature is about 78.9 K, which is well below the ITER design criterion. The maximum helium pressure inside the 316LN jacket is about 4.2 MPa, which is well below the ITER design criterion of 25 MPa.

**Fig. 10:**
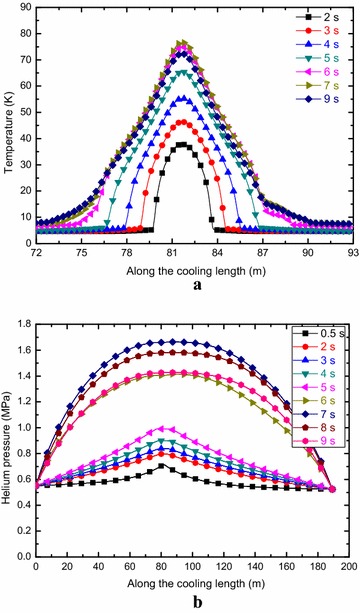
Cable temperature (**a**) and Helium pressure (**b**) evolution along the cooling length as functions of time for the B1 channel

Figure 10 shows the evolution of the cable temperature and helium pressure inside the jacket of the B1 channel along the cooling length for different times with the holding time of 2.0 s, initial disturbance length of 1 m and quench threshold voltage of 0.4 V. It is shown that the maximum cable temperature of the NbTi cable is about 76.7 K, which is well below the ITER design criterion. The maximum helium pressure inside the 316L jacket is about 1.66 MPa, which is well below the ITER design criterion of 25 MPa.

To evaluate the sensitivity of the maximum cable temperature and maximum helium pressure inside the jacket to the holding time, the initial disturbance length, and the threshold voltage, the parametric analysis was performed for both channels. The analysis results are shown in Tables 2, 3 and 4. The maximum cable temperature increases with increasing the holding time and threshold voltage for both channels. The disturbance length has the opposite effects on the maximum cable temperature. The threshold voltage and the holding time have a negligible impact on the maximum helium pressure while the initial disturbance length has a substantial impact on the maximum helium pressure. The maximum helium pressure is about 7.22 MPa for the threshold voltage of 0.4 V, the holding time of 2.0 s and the disturbance length of 10.0 m for the A1 channel. The maximum cable temperature is about 106.4 K for the threshold voltage of 0.4 V, the holding time of 4.0 s and the disturbance length of 1.0 m for the A1 channel. The maximum cable temperature is about 111.1 K for the threshold voltage of 0.4 V, the holding time of 4.0 s and the disturbance length of 1.0 m for the B1 channel. Therefore, the superconducting magnet can be protected with the threshold voltage of 0.4 V and holding time of 2.0 s.

**Table 2:** Sensitivity of maximum cable temperature and maximum helium pressure to disturbance length for the A1 and B1 channels

Disturbance length (m)	A1 channel		B1 channel	
	Max. cable temperature (K)	Max. helium pressure (MPa)	Max. cable temperature (K)	Max. helium pressure (MPa)
0.5	84.0	4.17	82.8	1.63
1.0 (Ref.)	78.9	4.18	76.6	1.66
10.0	58.0	7.22	50.3	2.93

**Table 3:** Sensitivity of maximum cable temperature and maximum helium pressure to threshold voltage for the A1 and B1 channels

Threshold voltage (V)	A1 channel		B1 channel	
	Max. cable temperature (K)	Max. helium pressure (MPa)	Max. cable temperature (K)	Max. helium pressure (MPa)
0.3	74.3	4.18	68.3	1.65
0.4 (Ref.)	78.9	4.19	76.6	1.66
0.45	81.3	4.22	82.3	1.68
0.50	83.8	4.24	82.4	1.69

**Table 4:** Sensitivity of maximum cable temperature and maximum helium pressure to holding time for the A1 and B1 channels

Holding time (s)	A1 channel		B1 channel	
	Max. cable temperature (K)	Max. helium pressure (MPa)	Max. cable temperature (K)	Max. helium pressure (MPa)
2.0 (Ref.)	78.9	4.18	76.6	1.66
2.5	85.0	4.19	83.9	1.70
3.0	91.6	4.22	92.0	1.75
3.5	98.7	4.24	101.0	1.81
4.0	106.4	4.27	111.1	1.90

## Conclusion

The temperature margin behavior and quench propagation behavior of the superconducting magnet are analyzed. The analysis results show that the magnet has the sufficient minimum temperature margin. The quench analysis is shown that the hot spot temperature and the helium pressure inside the jacket are well below the ITER design criteria. Therefore, the quench protection parameters selected can be designed to safely protect the superconducting magnet.
